# Time-Series Laplacian Semi-Supervised Learning for Indoor Localization †

**DOI:** 10.3390/s19183867

**Published:** 2019-09-07

**Authors:** Jaehyun Yoo

**Affiliations:** Department of Electrical, Electronic and Control Engineering, Hankyong National University, Anseoung 17579, Korea; jhyoo@hknu.ac.kr

**Keywords:** Wi-Fi RSSI-based indoor localization, semi-supervised learning, time-series learning

## Abstract

Machine learning-based indoor localization used to suffer from the collection, construction, and maintenance of labeled training databases for practical implementation. Semi-supervised learning methods have been developed as efficient indoor localization methods to reduce use of labeled training data. To boost the efficiency and the accuracy of indoor localization, this paper proposes a new time-series semi-supervised learning algorithm. The key aspect of the developed method, which distinguishes it from conventional semi-supervised algorithms, is the use of unlabeled data. The learning algorithm finds spatio-temporal relationships in the unlabeled data, and pseudolabels are generated to compensate for the lack of labeled training data. In the next step, another balancing-optimization learning algorithm learns a positioning model. The proposed method is evaluated for estimating the location of a smartphone user by using a Wi-Fi received signal strength indicator (RSSI) measurement. The experimental results show that the developed learning algorithm outperforms some existing semi-supervised algorithms according to the variation of the number of training data and access points. Also, the proposed method is discussed in terms of why it gives better performance, by the analysis of the impact of the learning parameters. Moreover, the extended localization scheme in conjunction with a particle filter is executed to include additional information, such as a floor plan.

## 1. Introduction

Wi-Fi received signal strength indicator (RSSI) is one of the basic sensory observations widely used for indoor localization. Due to its nonlinear and random properties, many machine learning approaches have been applied to Wi-Fi RSSI localization [1,2,3,4,5]. In particular, semi-supervised learning algorithms have been suggested to improve the efficiency, which reduces the human effort necessary for calibrating training data. For example, a large amount of unlabeled data can be easily collected by recording only Wi-Fi RSSI measurements, without assigning position labels, which can save resources for collection and calibration. By contrast, labeled training data must be created manually. Adding a large amount of unlabeled data in the semi-supervised learning framework can prevent the decrement in localization accuracy when using a small amount of labeled data.

The majority of the existing semi-supervised learning algorithms use the unlabeled data for the manifold regularization that captures the intrinsic geometric structure of the whole training data [6,7,8,9,10]. More progressed usage of unlabeled data is the pseudolabeling where unlabeled data are artificially labeled, and it is used for learning the estimation model. In [11,12,13], pseudolabels are iteratively updated, and the localization model is learned by the final pseudolabels. In [14], usage of unlabeled data avoids the biased parameter estimation against a small number of labeled data points, to obtain the probabilistic location-RSSI model. In [15,16], unlabeled data are used to find an embedding function from RSSI signal space to 2D location space. Semi-supervised deep learning approaches [17,18,19,20] improve positioning accuracy by using the unlabeled data for extracting features from the RSSI measurements in deep neutral network framework. Compared to discriminative model such as support vector machine (SVM); however, the deep learning methods normally take much time to finish the learning.

This paper proposes a new semi-supervised learning algorithm by interpreting the unlabeled data as spatio-temporal data. In the previous paper [21], the older version of the semi-supervised learning algorithm was applied to a mobile robot in a room size toy experiment. In this paper, the scalable algorithm has been developed for public indoor localization. The algorithm has two separate pseudolabeling and learning processes. First, in the pseudolabeling process, the time-series graph Laplacian SVM optimization is constructed, which estimates labels of the unlabeled data. The existing Laplacian SVM algorithms [6,7,8,9,10] consider only spatial relationship for the training data. To add the temporal meaning to the unlabeled data, this paper attaches the time-series regularization into the Laplacian SVM framework. Time-series learning is reasonable for the indoor localization targeting a smoothly moving human, and the corresponding data comes in a chronological order. As a result, the accurate pseudolabels are made by the proposed time-series semi-supervised learning.

Second, in the learning process, another learning framework to estimate position is constructed. Because the pseudolabels are artificial, the pseudolabeled data cannot be reliable as much as the labeled data. Therefore, it is desirable to limit the reliance on the pseudolabeled data. This can be dealt with a balancing optimization by weighting different balancing parameters between the labeled and pseudolabeled data. The existing semi-supervised methods [22], Ref. [11] might address this balance issue. In [22], the optimization framework consists of a singular value decomposition, a manifold regularization, and a loss function. In [11], the intermediate variable is introduced as a substitute of the original labeled data based on the graph Laplacian optimization framework. However, because many balancing parameters in [22], Ref. [11] are coupled to both the labeled and the unlabeled (or pseudolabeled) terms, it is difficult to adjust the balance. Also, as fewer labeled data are used, the pseudolabels become inaccurate. The proposed method solves this imbalance problem by adding Laplacian Least Square (LapLS) that is produced in this paper by combining the manifold regularization into the transductive SVM (TSVM [23]) structure. Because the pseudolabels are used as the learning input of LapLS, the proposed optimization becomes a linear problem that can be solved fast. In this learning process, two decoupled balancing parameters are individually weighed to the labeled term and the pseudolabeled term, separately, which makes it simple to balance the labeled and the pseudolabeled data. This balancing optimization adjusts the reliance on the pseudolabeled data relative to the labeled data. Outstanding performance of the proposed method is found even when a small amount of labeled training data are used.

The proposed algorithm is evaluated to estimate location of a smartphone carried by a user, and it is compared with some SVM-oriented semi-supervised algorithms. The accurate performance is validated by the analysis of the impact of the learning parameters. Also, according to the variation of the number of the labeled training data and the Wi-Fi access points, the proposed algorithm gives the best localization performance without sacrificing the computation time. In addition, the combination of the proposed learning-based estimation and the particle filter is implemented.

This paper is organized as follows. Section 2 overviews the learning-based indoor localization problem with description of experimental setup for Wi-Fi RSSI use. Section 3 presents the semi-supervised learning algorithms based on the graph Laplacian manifold learning. Section 4 devotes to introduce the proposed algorithm. Section 5 reports empirical results with the parameter setting, the localization with the variance of the number of the training data and access points, the computational time, and the filter-combined localization. Finally, concluding remarks are given in Section 6.

## 2. Learning-Based Indoor Localization

**Figure 1:**
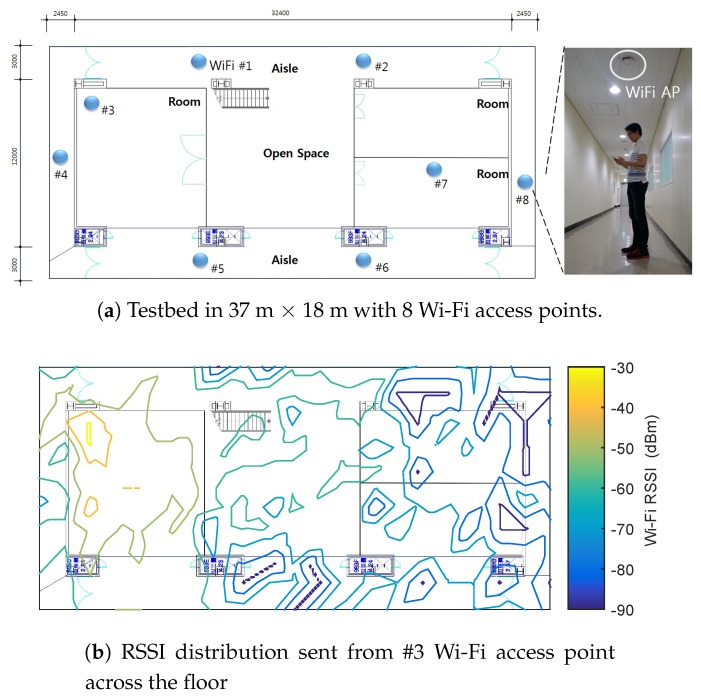

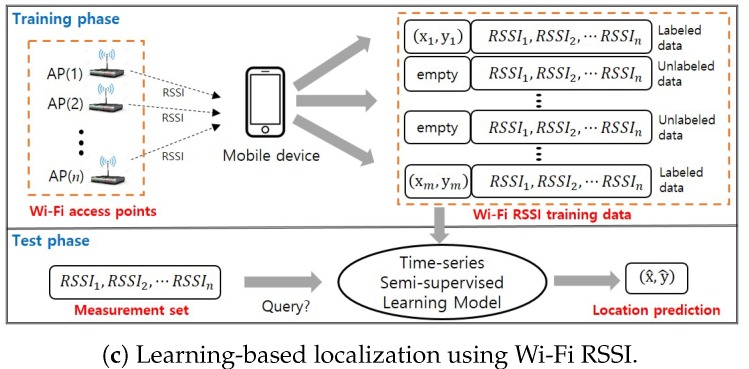
(**a**) Experimental floor with the 8 visible Wi-Fi access points, (**b**) Wi-Fi RSSI distribution of the #3 access point across the floor, and (**c**) learning-based localization architecture using Wi-Fi RSSI.

Figure 1a shows the localization setup where 8 Wi-Fi access points (APs) are deployed in the 37 × 18 $m^{2}$ floor. The APs are pre-installed by Korean telecommunication companies such as SKT and LGT. By distinguishing APs’ unique MAC (media access control) addresses, it can define a concentrated set by assigning each RSSI measurement sent from the different APs to the specific location in the set. The experimental floor consists of 3 rooms, open space, and aisle. Each room is filled with desks and chairs, and there are some obstacles such as a vase and a rubbish bin in places on the floor. 5 different people join to collect the training data. In case of collecting the labeled data, the trainers are guided to record the labeled data points on every 1.5 × 1.5 $m^{2}$ grid. During the calibration, the repeated measurement sets whose labels are the same location are averaged. As a result, 283 labeled training data are obtained. Similarly, another user is employed to collect 283 labeled *test* data, which are not used for any training algorithm. The smartphone device is Samsung Galaxy S7 operated by Android OS. The smartphone application to measure the Wi-Fi RSSIs and the locations is developed by Java Eclipse. The Wi-Fi scan function in the Android platform provides the information of MAC address and name of AP, and the RSSI value in dBm. Also, in this experiment, only 2.4 GHz RSSI signal can be collected.

Labeled training data are obtained by placing the receiver at different locations. Let us define the Wi-Fi observation set as $x_{i}=\left\{z_{i1},\ldots ,z_{in}\right\}\in R^{n}$ received from *n* different APs (n=8 in this paper), where $z_{ij}$ (1≤j≤n) is a scalar RSSI measurement sent from the *j*-th access point corresponding to the user’s location $(y_{Xi}$, $y_{Yi})$. Total of the *l* labeled training data are given by ${\left\{x_{i}\right\}}_{i=1}^{l}$ with $x_{i}\in X\subseteq R^{n}$, and ${\left\{y_{Xi}\right\}}_{i=1}^{l},{\left\{y_{Yi}\right\}}_{i=1}^{l}$. The unlabeled data set ${\left\{x_{i}\right\}}_{i=l+1}^{l+u}$ consists of only the RSSI measurements, without position labels. The training phase builds separate mappings $f_{X}:X\to R$ and $f_{Y}:X\to R$ which refer to relationships between Wi-Fi signal strength and location, using the labeled training data ${\left\{\left(x_{i},y_{Xi}\right)\right\}}_{i=1}^{l}$ and ${\left\{\left(x_{i},y_{Yi}\right)\right\}}_{i=1}^{l}$, respectively, and the unlabeled data ${\left\{x_{i}\right\}}_{i=l+1}^{l+u}$. Because the models $f_{X}$ and $f_{Y}$ are learned independently, we omit the subscripts of $f_{X}$, $f_{Y}$, and $y_{X}$, $y_{Y}$, for simplification.

Figure 1c illustrates overview of the proposed indoor localization architecture using Wi-Fi RSSI. The main purpose of the semi-supervised learning-based localization is to learn the accurate positioning model even when a small amount of labeled data is used. For example, Figure 1b shows the Wi-Fi RSSI distribution of the access point #3 that is in a room. Contribution of the semi-supervised learning is to prevent the distribution from being distorted when using much less labeled training data. This will be restated in Example 1 of Section 4.1.

## 3. Semi-Supervised Learning

This section describes basic semi-supervised learning framework in Section 3.1 and reviews Laplacian least square SVR (LapLS) in Section 3.2 and Laplacian embedded regression least square (LapERLS) in Section 3.3. Key ideas from these algorithms will be applied for the proposed algorithm in the next Section 4.

### 3.1. Basic Semi-Supervised SVM Framework

Given a set of *l* labeled samples ${\left\{\left(x_{i},y_{i}\right)\right\}}_{i=1}^{l}$ and a set of *u* unlabeled samples ${\left\{x_{i}\right\}}_{i=l+1}^{l+u}$, Laplacian semi-supervised learning aims to establish a mapping *f* by the following regularized minimization functional [24]:

(1) $$f^{*}=\arg\min_{f\in H_{k}}C\sum_{i}V\left(x_{i},y_{i},f\right)+\gamma_{A}{∥f∥}_{A}^{2}+\gamma_{I}{∥f∥}_{I}^{2},$$

where *V* is a loss function, ${∥f∥}_{A}^{2}$ is the norm of the function in the Reproducing Kernel Hilbert Space (RKHS) $H_{k}$, ${∥f∥}_{I}^{2}$ is the norm of the function in the low-dimensional manifold, and *C*, $\gamma_{A}$, $\gamma_{I}$ are the regularization weight parameters.

The solution of (1) is defined as an expansion of kernel function over the labeled and the unlabeled data, given by

(2) $$f\left(x\right)=\sum_{i=1}^{l+u}\alpha_{i}K\left(x_{i},x\right)+b,$$

with the bias term *b* and the kernel function $K\left(x_{i},x_{j}\right)=\langle \phi \left(x_{i}\right),\phi \left(x_{j}\right)\rangle$, where ϕ(·) is a nonlinear mapping to RKHS.

The regularization term ${∥f∥}_{A}^{2}$ associated with RKHS is defined as

(3) $${∥f∥}_{A}^{2}={\left(\Phi \alpha \right)}^{T}\left(\Phi \alpha \right)=\alpha^{T}K\alpha ,$$

where $\Phi =[\phi \left(x_{1}\right),\ldots ,\phi \left(x_{l+u}\right)]$, $\alpha ={\left[\alpha_{1},\ldots ,\alpha_{l+u}\right]}^{T}$, and *K* is the (l+u)×(l+u) kernel matrix whose element is $K_{ij}$. We adopt Gaussian kernel given by

(4) $$K_{ij}=K\left(x_{i},x_{j}\right)=\exp\left(-∥x_{i}-x_{j}{∥}^{2}/\sigma_{k}^{2}\right),$$

where $\sigma_{k}^{2}$ is the kernel width parameter.

According to the manifold regularization, data points are samples obtained from a low-dimensional manifold embedded in a high-dimensional space. This is represented by the graph Laplacian:

(5) $$\begin{array}{ccc} {∥f∥}_{I}^{2} & = & \frac{1}{{\left(l+u\right)}^{2}}\sum_{i=1}^{l+u}\sum_{j=1}^{l+u}W_{ij}{\left(f\left(x_{i}\right)-f\left(x_{j}\right)\right)}^{2},\\ & = & \frac{1}{{\left(l+u\right)}^{2}}f^{T}Lf, \end{array}$$

where *L* is the normalized graph Laplacian given by $L=D^{-1/2}\left(D-W\right)D^{-1/2}$, $f={\left[f\left(x_{1}\right),\ldots ,f\left(x_{l+u}\right)\right]}^{T}$, *W* is the adjacency matrix of the data graph, and *D* is the diagonal matrix given by $D_{ii}=\sum_{j=1}^{l+u}W_{ij}$. In general, the edge weights $W_{ij}$ are defined as Gaussian function of Euclidean distance, given by

(6) $$W_{ij}=\exp\left(-∥x_{i}-x_{j}{∥}^{2}/\sigma_{w}^{2}\right),$$

where $\sigma_{w}^{2}$ is the kernel width parameter.

Minimizing ${∥f∥}_{I}^{2}$ is equivalent to penalizing the rapid changes of the regression function evaluated between two data points. Therefore, $\gamma_{I}{∥f∥}_{I}^{2}$ in (1) controls the smoothness of the data geometric structure.

### 3.2. Laplacian Least Square (LapLS)

This section describes developing of the LapLS algorithm algorithm by combining manifold regularization defined in (5) and transductive SVM (TSVM) [23]. In LapLS, the loss function *V* in (1) is defined by

(7) $$V\left(x_{i},y_{i},f\right)=e_{i}=y_{i}-\left(\sum_{i=1}^{l+u}\alpha_{i}K\left(x_{i},x\right)+b\right).$$

LapLS finds optimal parameters α, *b*, and the labels $y_{1}^{*},\ldots ,y_{u}^{*}$ of the unlabeled data when regularization parameters *C* and $C^{*}$ are given:

(8) $$\begin{array}{c} \min_{\alpha ,e,e^{*},b,y_{1}^{*},\ldots ,y_{u}^{*}}\frac{C}{2}\sum_{i=1}^{l}e_{i}^{2}+\frac{C^{*}}{2}\sum_{j=1}^{u}{\left(e_{j}^{*}\right)}^{2}+\gamma_{A}\alpha^{T}K\alpha +\gamma_{I}\alpha^{T}KLK\alpha ,\\ \mathrm{subject}\;\mathrm{to}:y_{i}-\sum_{k=1}^{l+u}\alpha_{k}K_{ik}-b-e_{i}=0,\;\;i=1,\ldots ,l.\\ y_{j}^{*}-\sum_{k=1}^{l+u}\alpha_{k}K_{jk}-b-e_{j}^{*}=0,\;\;j=1,\ldots ,u. \end{array}$$

Optimization in (8) with respect to all $y_{1}^{*},\ldots ,y_{u}^{*}$ is a combinatorial problem [23]. To find the solution, we must search over all possible $2^{u}$ labels of the unlabeled data. Therefore, this method is not useful when a large amount of the unlabeled data is applied.

### 3.3. Laplacian Embedded Regularized Least Square (LapERLS)

LapERLS introduces an intermediate decision variable $g\in R^{\left(l+u\right)}$ and additional regularization parameter $\gamma_{C}$ into the Laplacian semi-supervised framework (1), as follows [11]:

$$\begin{array}{c} \min_{f\in H_{k},g\in R^{\left(l+u\right)}}C\sum_{i=1}^{l+u}V\left(x_{i},g_{i},f\right)+\gamma_{C}\sum_{i=1}^{l}{\left(g_{i}-y_{i}\right)}^{2}+\gamma_{A}{∥f∥}_{A}^{2}+\gamma_{I}{∥g∥}_{I}^{2}. \end{array}$$

The optimization problem of (9) enforces the intermediate decision variable *g* to be close to the labeled data and to be smooth with respect to the graph manifold.

Loss function is given by:

(9) $$V\left(x_{i},g_{i},f\right)=\xi_{i}=g_{i}-\left(\sum_{j=1}^{l+u}\alpha_{j}K\left(x_{i},x_{j}\right)\right).$$

After reorganizing the terms in (9) with respect to manifold regularization and decision function and corresponding parameter, the primal optimization problem is as follows:

(10) $$\begin{array}{c} \min_{\alpha ,g,\xi \in R^{\left(l+u\right)}}\frac{C}{2}\sum_{i=1}^{l+u}\xi_{i}^{2}+\alpha^{T}K\alpha +\frac{1}{2}{\left(g-y\right)}^{T}\Lambda \left(g-y\right)+\frac{1}{2}\mu g^{T}Lg,\\ \mathrm{subject}\;\mathrm{to}:\xi_{i}=g_{i}-\sum_{k=1}^{l+u}\alpha_{k}K_{ik},\;\;\;i=1,\ldots ,l+u, \end{array}$$

where Λ is a diagonal matrix of trade-off parameters with $\Lambda_{ii}=\lambda$ if $x_{i}$ is a labeled data point, and $\Lambda_{ii}=0$ if $x_{i}$ is unlabeled, $y={\left[y_{1},\ldots ,y_{l},0,\ldots ,0\right]}^{T}\in R^{\left(l+u\right)}$, and *C*, λ, μ are tuning parameters.

Also, dual formulation of (10) is given by:

(11) $$\begin{array}{c} \min_{\beta \in R^{\left(l+u\right)}}\frac{1}{2}\beta^{T}\tilde{Q}\beta +\beta^{T}\tilde{y}, \end{array}$$

where

(12) $$\begin{array}{ccc} \tilde{Q} & = & K+{\left(\Lambda +\mu L\right)}^{-1},\\ \tilde{y} & = & {\left(\Lambda +\mu L\right)}^{-1}\Lambda y,\\ \beta & = & -\alpha . \end{array}$$

The main characteristics of this method lies in using $\tilde{y}$ as the input to learning, unlike standard semi-supervised learning that uses $y={\left[y_{1},\ldots ,y_{l},0,\ldots ,0\right]}^{T}$. In other words, zero values in *y* are modified to some values denoting pseudolabels of unlabeled data.

It is noted that when the original labeled set *y* is replaced with the intermediate decision variable *g*, the accuracy of the learning may decrease. Moreover, when amount of available labeled data is small, the accuracy of the pseudolabels, which is difference between true labels of unlabeled data points and pseudolabels of the unlabeled data points, decreases significantly.

## 4. Time-Series Semi-Supervised Learning

This section describes a new algorithm by extracting key ideas from LapLS and LapERLS introduced in the previous section. In Section 4.1, a time-series representation is added to the unlabeled data to obtain pseudolabels. In Section 4.2, the pseudolabels are used in LapLS structure to derive an optimal solution by balancing the pseudolabeled and the labeled data. Notations are equivalent to the previous section.

### 4.1. Time-Series LapERLS

In [25], a time-series learning optimization problem is suggested by applying Hodric-Prescott (H-P) filter [26], which can capture a smoothed-curve representation of a time-series from the training data, given by

(13) $$\min_{f}\sum_{i=1}^{t}{\left(f\left(x_{i}\right)-y_{i}\right)}^{2}+\gamma_{T}\sum_{i=3}^{t}{\left(f\left(x_{i}\right)+f\left(x_{i-2}\right)-2f\left(x_{i-1}\right)\right)}^{2},$$

where ${\left\{\left(x_{i},y_{i}\right)\right\}}_{i=1}^{t}$ is the time-series labeled training data. The second term is to make the sequential points $f\left(x_{i}\right),f\left(x_{i-1}\right),f\left(x_{i-2}\right)$ on a line. The solution of (13) in the matrix form is,

$$f={\left(I+\gamma_{T}DD^{T}\right)}^{-1}y,$$

where

(14) $$\begin{array}{c} D={\left[\begin{array}{ccccccc} 0 & 0 & \cdots & \cdots & \cdots & \cdots & 0\\ 0 & 0 & 0 & \cdots & \cdots & \cdots & 0\\ 1 & -2 & 1 & 0 & \cdots & \cdots & 0\\ 0 & 1 & -2 & 1 & 0 & \cdots & 0\\ \vdots & \vdots & \vdots & \vdots & \vdots & \vdots & \vdots \\ 0 & \cdots & \cdots & 0 & 1 & -2 & 1 \end{array}\right]}_{t\times t\;.} \end{array}$$

The main idea for a new semi-supervised learning is to assign additional temporal meaning to the unlabeled data. In the proposed algorithm, the H-P filter is added into the LapERLS formulation (9) in the following optimization:

(15) $$\begin{array}{c} \min_{f\in H_{k},g\in R^{\left(l+u\right)}}C\sum_{i=1}^{l+u}V\left(x_{i},g_{i},f\right)+\gamma_{C}\sum_{i=1}^{l}{\left(g_{i}-y_{i}\right)}^{2}+\gamma_{A}{∥f∥}_{A}^{2}+\gamma_{I}{∥g∥}_{I}^{2}\\ +\gamma_{T}\sum_{i=3}^{l+u}{\left(g\left(x_{i}\right)+g\left(x_{i-2}\right)-2g\left(x_{i-1}\right)\right)}^{2}. \end{array}$$

After rearranging (15) using the process similar to Equations (9)–(11), the optimization form of the proposed time-series LapERLS is given by:

(16) $$\begin{array}{c} \min_{\beta \in R^{\left(l+u\right)}}\frac{1}{2}\beta^{T}\tilde{Q}\beta +\beta^{T}\tilde{y}, \end{array}$$

where

(17) $$\begin{array}{ccc} \tilde{Q} & = & K+{\left(\Lambda +\mu_{1}L+\mu_{2}DD^{T}\right)}^{-1}, \end{array}$$

(18) $$\begin{array}{ccc} \tilde{y} & = & {\left(\Lambda +\mu_{1}L+\mu_{2}DD^{T}\right)}^{-1}\Lambda y, \end{array}$$

$$\begin{array}{ccc} \beta & = & -\alpha . \end{array}$$

In comparison with (12) of the standard LapERLS, $\mu_{2}DD^{T}$ is added to (18). In the following, the experimental examples for describing the difference of the standard LapERLS and the time-series LapERLS are introduced.

**Figure 2:**
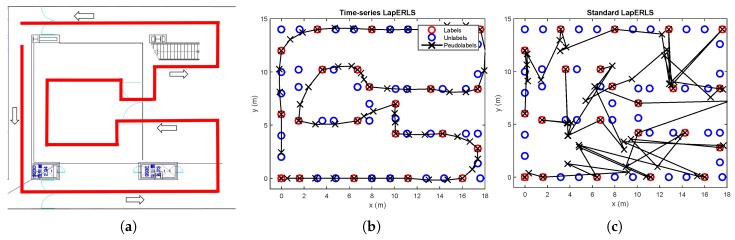
Comparison of accuracy of pseudolabels between time-series LapERLS (18) and standard LapERLS (12). The labeled, unlabeled, and pseudolabeled data points are marked by red circles, blue circles, and black crosses, respectively. (**a**) A user follows the red path and collects the labeled and unlabeled data. (**b**) The resultant pseudolabels from time-series LapERLS are so accurate that the trajectory made by the pseudolabels is close to the user’s true path. (**c**) Due to inaccurate pseudolabels obtained from the standard LapERLS, this trajectory is severely incorrect.

**Example** **1.** *Figure 2 examines the accuracy comparison between the standard LapERLS and the time-series LapERLS. In this example, a time-series training data set is collected as a user moves along the path illustrated in Figure 2a. This simulation uses 20% of the labeled training data and 80% of the unlabeled data among total 283 training data. Figure 2 illustrates estimations of the pseudolabels using each time-series LapERLS and standard LapERLS. As shown in Figure 2b,c, the pseudolabels produced by the time-series LapERLS are accurate while the standard LapERLS does not show meaningful pseudolabels. Therefore, the trajectory of pseudolabels from the time-series LapERLS can recover the true trajectory while the standard LapERLS cannot. Obviously, many incorrect pseudolabels such as Figure 2c will derive inaccurate localization performance.*

**Figure 3:**
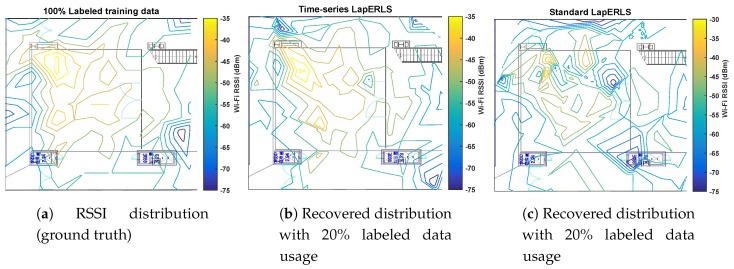
Comparison of the Wi-Fi RSSI distributions made by the pseudolabels between the time-series LapERLS (proposed) and the standard LapERLS (compared). In (**a**), the original RSSI distribution sent from #3 access point marked in Figure 1 is given as the ground truth and is made by using the entire labeled training data. In (**b**), the time-series LapERLS algorithm with usage of 20% of the labeled training data produces the similar distribution to the original distribution due to the accurately estimated pseudolabels. In (**c**), the standard LapERLS with usage of 20% labeled training data produces the severely distorted distribution.

*The other physical interpretation about the pseudolabels can be seen from Figure 3. Figure 3a shows the RSSI distribution as the ground truth by using the entire labeled training data, Figure 3b shows the RSSI distribution recovered by the pseudolabels obtained by the time-series LapERLS, and Figure 3c is the RSSI distribution estimated by the pseudolabels of the standard LapERLS. In case of the standard LapERLS as shown in Figure 3c, the distribution is severely distorted due to the incorrectly estimated pseudolabels. On the other hand, the time-series LapERLS gives the very similar distribution to the original distribution.*

**Example** **2.** *Accuracy of the pseudolabels is examined by the three cases with comparison to a linear interpolation.*

**Figure 4:**
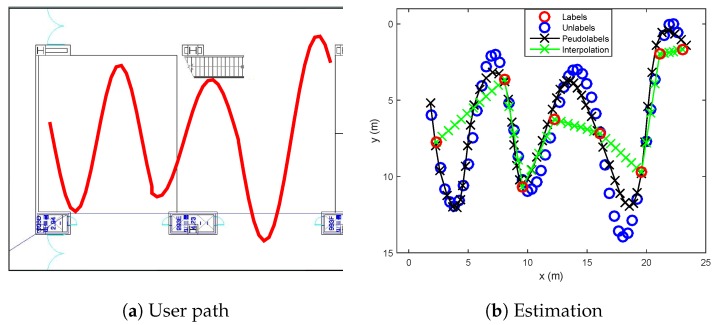
Estimated pseudolabels on the sinusoidal trajectory in comparison with the linear interpolation.

**Figure 5:**
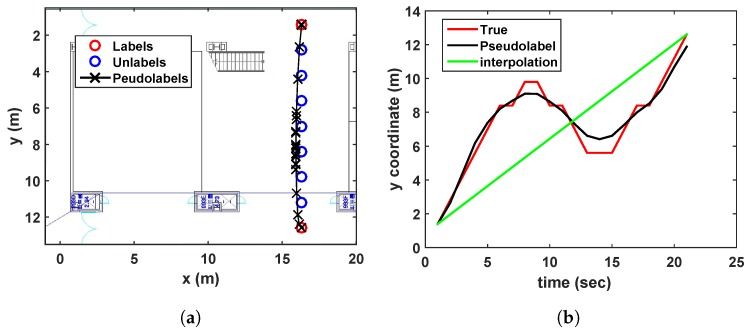
Estimated pseudolabels on the wandered trajectory in (**a**), in comparison with the linear interpolation in (**b**).

**Figure 6:**
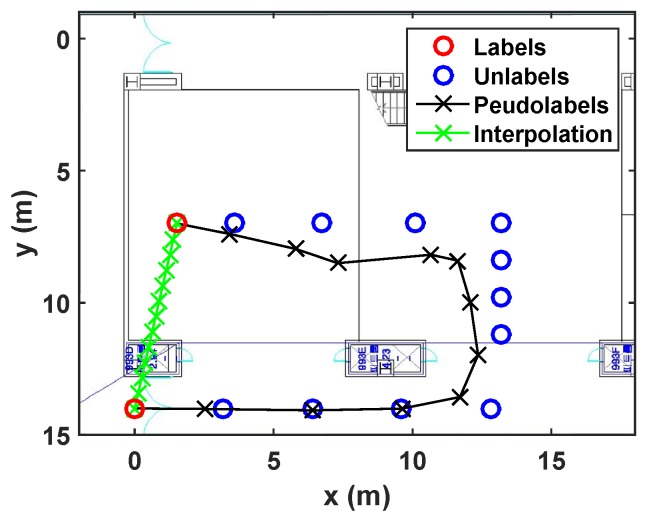
Estimated pseudolabels when revisiting the learned trajectory in comparison with the linear interpolation.

- Sinusoidal trajectory:
  A situation that a user moves a sinusoidal trajectory as described in Figure 4a is considered. The linear interpolation produces the pseudolabels laying on the straight line between two consecutive labeled points. On the other hand, because the proposed algorithm considers both spatial and temporal relation by using manifold and time-series learning, the result of the suggested algorithm can generate accurate pseudolabels as shown in Figure 4b.
- Wandered trajectory:
  The other case in which the linear interpolation is not useful is when the user does not walk straight forward to a destination. For example, in Figure 5, the user wanders around middle of the path. Because there are only two labeled data (one is at bottom, and the other is at top), the linear interpolation could not represent the wandered trajectory. In Figure 5b, it is shown that the developed algorithm generates accurate pseudolabels with respect to the wandered motion of the user.
- Revisiting the learned trajectory:
  The user used to revisit the same site during collecting training data. Suppose that the locations corresponding to the Wi-Fi RSSI measurements are not recorded during walking a path, except the start and end points as shown in Figure 6. It is assumed that we have already learned those area. The result under this situation is shown in Figure 6, where the developed algorithm generates accurate pseudolabels, while the linear interpolation cannot reflect the reality.

### 4.2. Balancing Labeled and Pseudolabeled Data

Regardless of how accurate the pseudolabels are, it is impossible to regard the pseudolabels as the labeled data, because true labels of the unlabeled data are unknown. One desirable approach is to properly balance the labeled and the pseudolabeled data in the learning process. This is feasible by applying LapLS structure in Section 3.2, which can control the balance of training data by the decoupled parameters *C* and $C^{*}$ introduced in (8).

**Figure 7:**
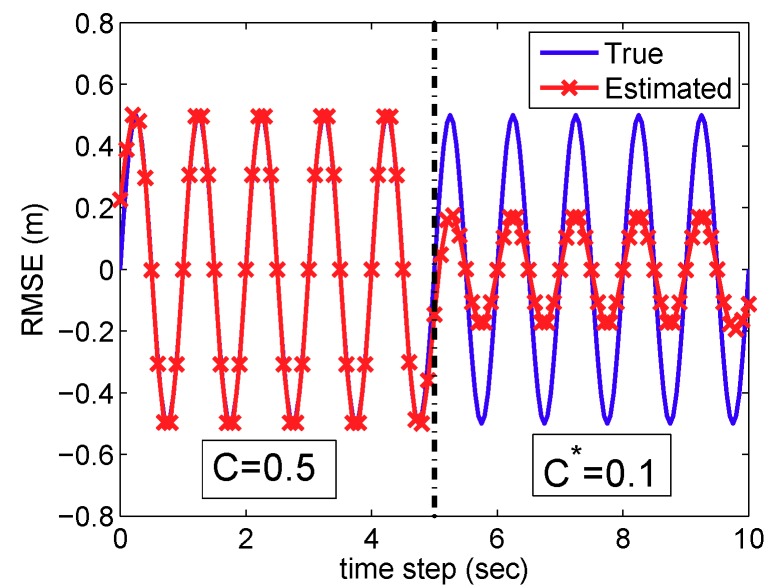
Sine function estimation using LapLS in Section 3.2 with different values of balancing parameters *C* and $C^{*}$.

**Example** **3.** Figure 7 illustrates an estimation of the sine function using LapLS in Section 3.2, where we divide the labeled training set in half and use the different values of the parameters, i.e., C=0.5 and $C^{*}=0.1$. In the latter part, the estimation with $C^{*}=0.1$ is not accurate. This result can be validated from (8). As the parameter $C^{\left(*\right)}$ becomes smaller, the related term $C^{\left(*\right)}\sum_{i}{\left(e_{i}^{\left(*\right)}\right)}^{2}$ becomes also smaller. In other words, the optimization focuses less on the training data points with the smaller parameter value of $C^{\left(*\right)}$.

For the final step in the suggested algorithm, the idea is to treat pseudolabels $\tilde{y}$ in (18) as the labels of the unlabeled data in the LapLS (8) framework, which forms the following optimization:

(19) $$\begin{array}{c} \min_{\alpha \in R^{\left(l+u\right)},e\in R^{l},e^{*}\in R^{u},b\in R}\frac{C}{2}\sum_{i=1}^{l}e_{i}^{2}+\frac{C^{*}}{2}\sum_{j=1}^{u}{\left({\tilde{e}}_{j}^{*}\right)}^{2}+\gamma_{A}\alpha^{T}K\alpha +\gamma_{I}\alpha^{T}KLK\alpha ,\\ \mathrm{subject}\;\mathrm{to}:y_{i}-\sum_{k=1}^{l+u}\alpha_{k}K_{ik}-b-e_{i}=0,\;\;i=1,\ldots ,l.\\ {\tilde{y}}_{j}^{*}-\sum_{k=1}^{l+u}\alpha_{k}K_{jk}-b-{\tilde{e}}_{j}^{*}=0,\;\;j=1,\ldots ,u, \end{array}$$

where ${\tilde{y}}_{j}^{*}$ are pseudolabels of the unlabeled data from $\tilde{y}$ (18). Therefore, the non-convex problem of LapLS (8) is modified to a convex problem due to insertion of the pseudolabels. After KKT conditions, we obtain the following linear system:

(20) AX=Y,

with

$$\begin{array}{ccc} A & = & \left[\begin{array}{cc} K+\Gamma & 1_{(l+u)\times 1}\\ 1_{1\times (l+u)} & 0 \end{array}\right],\\ X & = & \left[\begin{array}{c} \alpha \\ b \end{array}\right],Y=\left[\begin{array}{c} \tilde{Y}\\ 0 \end{array}\right], \end{array}$$

where *K* is the kernel matrix in (4), $\alpha =\left[\alpha_{1},\ldots ,\alpha_{l+u}\right]\in R^{\left(l+u\right)}$, b∈R in (2) is the bias value, $1_{(l+u)\times 1}={\left[1,\ldots ,1\right]}^{T}\in R^{\left(l+u\right)}$ is the one vector, and Γ is the diagonal matrix with $\Gamma_{ii}=1/C$ for i=1,…,l and $\Gamma_{ii}=1/C^{*}$ for i=l+1,…,l+u. The pseudolabel vector $\tilde{Y}\in R^{\left(l+u\right)}$ is a time-series set of the labeled and pseudolabeled data. The optimal solution $\alpha^{*}$ and $b^{*}$ obtained after solving (20) becomes the final parameters of the localization model, which is defined in (2). In the test phase, when a user queries location by sending a RSSI measurement set, the location is estimated based on the learned localization model.

The proposed algorithm in (20) improves the performance of LapERLS by combining the structure of LapLS. First, the pseudolabels are accurately estimated by assigning temporal-spatio representation into the unlabeled data. Second, it is easy to adjust the balance between the pseudolabels and the labeled data. Moreover, by incorporating the pseudolabels into the LapLS structure, the non-convex problem is transformed to a convex problem that can be computed far faster. The proposed algorithm is summarized in Algorithm 1.

**Algorithm 1** Proposed semi-supervised learning for localization
Step 1:Collect labeled training data set ${\left\{\left(x_{i},y_{i}\right)\right\}}_{i=1}^{l}$ and unlabeled data set ${\left\{x_{j}\right\}}_{j=1}^{u}$ in a time-series.Step 2:Obtain kernel matrix *K* in (4), normalized Laplacian matrix *L* in (5) and matrix Λ in (10).Step 3:Choose values of $\mu_{1}$ and $\mu_{2}$ in (18), and then calculate the pseudolabels in (18).Step 4:Choose values of *C* and $C^{*}$, and then solve the linear equation in (20).Step 5:Based on the optimal solution $\alpha^{*}$ and $b^{*}$ from Step 4, builds the localization model in (2).


## 5. Experiments

To evaluate the proposed algorithm in comparison with other semi-supervised learning algorithms, two kinds of error are defined. First is the pseudolabel error defined as the average of the distance errors between unlabeled data and pseudolabeled data. Second is the test error is defined as the average of the distance errors between true locations and estimates.

This section consists of the parameter setting in Section 5.1 and the compared localization results according to the variation of the training data in Section 5.2. In Section 5.3, the result according to the variation of the number of Wi-Fi access points and the computation analysis are given. Finally, Section 5.4 describes a combination of particle filter and the suggested learning algorithm.

### 5.1. Parameter Setting

The hyper-parameters in machine learning algorithms are generally tuned by cross validation that selects parameters values to minimize training error of some split training data sets, e.g., 10-fold cross validation [27]. However, most semi-supervised learning applications use a small number of the labeled data, so it is not suitable to employ the cross validation. This section provides a guideline for selecting all the parameters used in the developed algorithm, by describing the physical meaning of each parameter.

**Figure 8:**
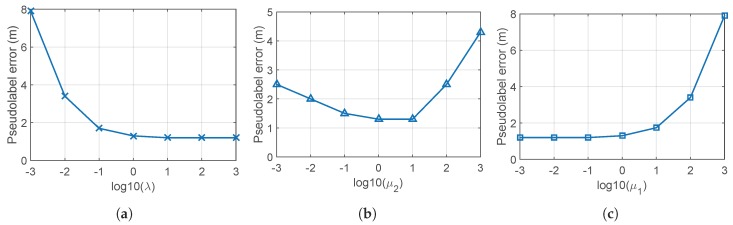
Impact of the parameters λ in Step 2, $\mu_{2}$, and $\mu_{1}$ in Step 3 of Algorithm 1. (**a**) Impact of the value λ when $\mu_{1}=1$, $\mu_{2}=1$. (**b**) Impact of the ratio $\mu_{2}/\mu_{1}$; we set $\mu_{1}=1$ and vary $\mu_{2}$. (**c**) Impact of the value of $\mu_{1}$ and $\mu_{2}$ if the ratio $\mu_{2}/\mu_{1}$ is fixed, we vary $\mu_{1}$ and $\mu_{2}$ with constraint $\mu_{2}/\mu_{1}=1$.

First, λ, i.e., the diagonal elements of Λ in Step 2 of Algorithm 1, can be interpreted as the importance of the labeled data relative to the unlabeled data by (12) and (18). If λ is relatively smaller than the particular value defined as $\lambda_{critical}$, resultant pseudolabels of the labeled data are different from the true labels. Therefore, it is decided to select value of λ larger than such critical value. Figure 8a shows the pseudolabel error according to the variation of λ, where $\lambda_{critical}=10$ becomes the proper parameter selection.

Second, selection of $\mu_{1}$ and $\mu_{2}$ in Step 3 of Algorithm 1 represents a trade-off relationship between spatial and temporal correlation. If it is desirable to weigh the temporal meaning more than the spatial meaning, $\mu_{2}$ is selected larger than $\mu_{1}$. Figure 8b shows the error of the pseudolabels according to the variation of $\mu_{2}$ at fixed $\mu_{1}$, which suggests that $1<\mu_{2}<10$ is proper for this data set. Also, Figure 8c gives the impact of the ratio $\mu_{2}/\mu_{1}$, where $\mu_{2}/\mu_{1}<1$ is proper to this data set.

**Figure 9:**
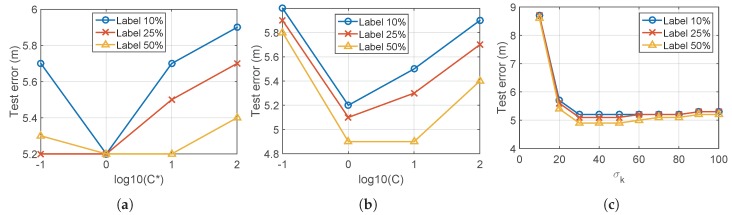
Impact of the parameters *C*, $C^{*}$, and $\sigma_{k}$ in Step 4 of Algorithm 1. Other parameters used in the prior Step 3 are set to $\mu_{1}=1$, $\mu_{2}=3$, and λ=5. (**a**) Impact of the ratio $C^{*}/C$ when C=40. (**b**) Impact of the values of *C* and $C^{*}$ if the ratio $C/C^{*}$ is fixed, varying *C* and $C^{*}$ with constraint $C/C^{*}=1$. (**c**) Impact of the value of $\sigma_{k}$ when C=40 and $C^{*}=40$.

Third, selection of *C* and $C^{*}$ in Step 4 of Algorithm 1 represents a trade-off relationship about the relative importance between the labeled data and the pseudolabeled data, as described in V-B. It is highlighted again that $C^{*}$ should be smaller than *C* if we want to reduce the reliance of the pseudolabeled data relative to the labeled data. We used 10%, 25%, and 50% labeled data points among total of 283 labeled data points to test an effect of the parameters *C* and $C^{*}$ whose results are shown in Figure 9a,b. From Figure 9a, it is observed that $C^{*}$ smaller than *C* can reduce the test error. In particular, the same test error is found at $C^{*}=1$, which implies that the algorithm with only 10% labeled data can have the same performance when using large amount of 50% used labeled data. This result highlights the importance of balancing the labeled and the pseudolabeled data and proves why the proposed algorithm can give good results when using a small number of the labeled data points. Also, Figure 9b shows the impact of values of *C* and $C^{*}$ when the ratio $C/C^{*}$ is fixed. It is shown that $10<C/C^{*}<50$ is proper for this data set. Lastly, Figure 9c shows the test error with respect to the variance of $\sigma_{k}$ used for the kernel matrix. Table 1. summarizes the parameter values used for the rest of the experiments.

### 5.2. Variation of Number of Training Data

**Figure 10:**
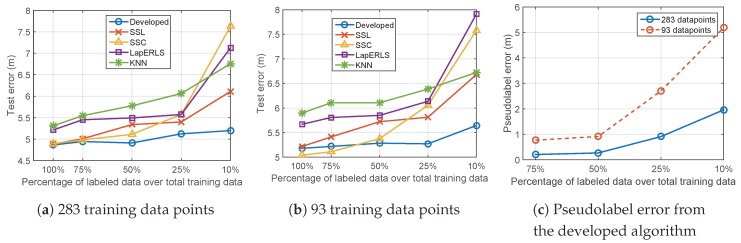
Localization results of the compared algorithms with respect to the variation of the ratio of the labeled training data over (**a**) total of 283 training data points and (**b**) total of 93 training data points. In (**c**), accuracy of the pseudolabels obtained from the developed algorithm is examined according to variation of the number of labeled data.

This experiments compares the semi-supervise algorithms, i.e., SSL [28], SSC [22], LapERLS [11] to the proposed algorithm with respect to the variation of the number of labeled training data at the fixed total of the training data, and the results are shown in Figure 10. For example, in case of 75% labeled data, 25% unlabeled data is used. The total of 283 and 93 training data points are used in Figure 10a,b, respectively. From the both results, we can observe that our algorithm outperforms the compared methods. In cases of 100% and 75% labeled data in Figure 10b, SSC gives slightly smaller error than the others. However, considering the advantage given to only SSC, i.e., the information of locations of the Wi-Fi access points, this error reduction is not noticeable.

The major contribution of our algorithm is found from the results when a small amount of labeled data is used. From Figure 10a,b, our algorithm shows the slightly increasing errors as less labeled data are used, while the others give the highly increasing error from 25% percentage. To analyze this result, we check the error of the pseudolabeled data points made in our algorithm, in Figure 10c. When using many labeled training data points such as 50%∼75%, accurate pseudolabels are made, so good learning result is obvious. Even though the pseudolabeled data is inaccurate such that only 10% labeled data is used as in Figure 10c, the test error is still small as in Figure 10a,b. This is because we balanced the pseudolabeled data by reducing the reliance on the pseudolabeled data relative to the labeled data.

### 5.3. Variation of Number of Wi-Fi Access Points and Computational Time

**Figure 11:**
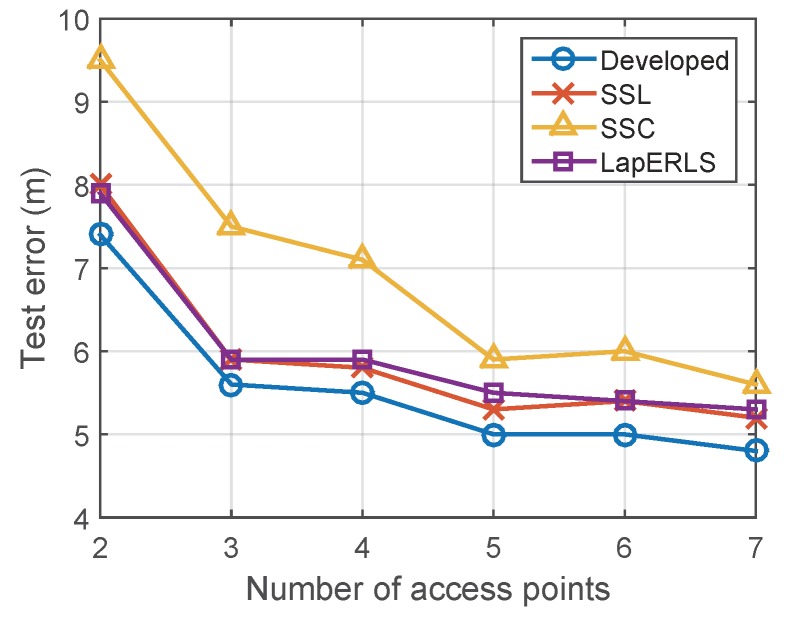
Impact of the number of Wi-Fi access points for localization performance.

Figure 11 compares the results when the number of Wi-Fi access points (AP) is changed from 2 to 7, where the APs depicted in Figure 1 are removed by this order #1 → #6 → #3 → #7 → #8 → #4.

**Figure 12:**
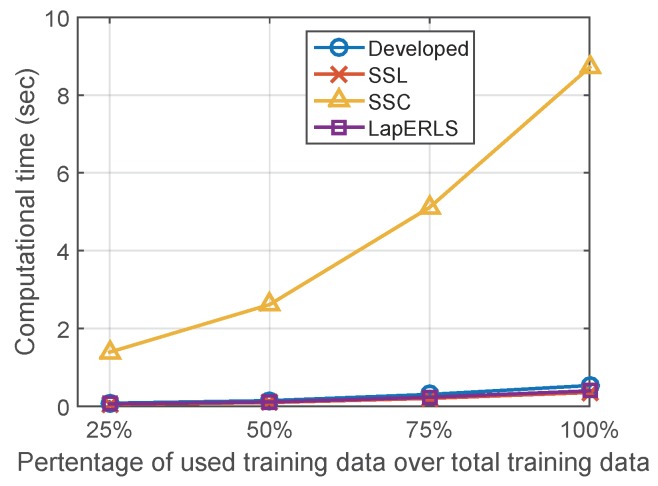
Computational time of the compared algorithms.

Figure 12 summarizes the computational training times of the compared algorithms. Computational complexity of the optimization of SSL, LapERLS, and the proposed algorithm is $O(dn^{2}+n^{3})$ [29] by the matrix computation and its inversion in (18), where *d* is the dimension of training data and *n* is the number of training data. In case of SSC, it additionally needs the computation of harmonic function using *k*-NN search algorithm which is O(nk(n+d)log(n+d)) [30]. The computation time of SSC increases significantly according to the increasing amount of the labeled data while the others remain lower-bounded in 0.5 s. The proposed algorithm needs little more time than LapERLS and SSL (at most 0.2 s).

### 5.4. Combination with Particle Filter

While the previous sections have shown the results of localization using the Wi-Fi RSSIs based on the learning algorithms, this section provides a combination with a filtering algorithm. An application of the particle filter can use other kinds of positioning information such as an IMU measurement and map information, to improve indoor localization. Because a detailed description about the particle filter can be found in many works [31,32,33], this paper simply describes how that information with the learning-based localization can be combined in the particle filter framework.

First, a dynamic model given an IMU sensor data can be defined by:

(21) $$\begin{array}{c} X_{t+1}=X_{t}+{\hat{V}}_{t}\Delta t\left[\begin{array}{c} \cos({\hat{\theta }}_{t})\\ \sin({\hat{\theta }}_{t}) \end{array}\right], \end{array}$$

where $X_{t}$ is a 2-D location at time step *t*, and ${\hat{V}}_{t}$ and ${\hat{\theta }}_{t}$ are the velocity and direction of an user, respectively. However, in (21), accurate estimation for the velocity and the direction is difficult to be obtained for the indoor positioning. For example, for obtaining accurate velocity, the attitude of the smartphone needs to be kept fixed without rotating. More specifically, the estimates of a step detection and velocity of the user have the significant error when the user swings the smartphone by walking. Also, the estimation of the direction is much biased in the indoor environment due to the ferrous material and the integral gyro error.

For such reasons, an alternative dynamic model without employing IMU measurement is the random walk defined by the Gaussian distribution as follows:

(22) $$X_{t+1}∼N\left(X_{t},\Sigma_{d}\right).$$

Next, the particle filter uses the estimated location obtained from the learning algorithm as the observation model in the following:

$$Z_{t}=HX_{t}+\Gamma ,$$

where $Z_{t}={\left[f_{X},f_{Y}\right]}^{T}$ is the estimated location from the learning algorithm $f_{X},\;f_{Y}$, and *H* is the identity matrix, and Γ is a Gaussian noise whose mean is zero and variance is $\Sigma_{o}$.

The key point of the particle filter is to update the weights $w_{t}^{i}$ of each particle $X_{t}^{i}$ for i=1,…,m, where *m* is the number of the particles. It decides the estimation ${\hat{X}}_{t}$ as the weighted summation ${\hat{X}}_{t}=\sum_{i=1}^{m}w_{t}^{i}X_{t}^{i}$. The weights are updated in the following:

$$w_{t}^{i}=w_{t-1}^{i}\cdot P\left(Z_{t}|X_{t}^{i}\right)\cdot P\left(X_{t}^{i}|X_{t-1}^{i}\right),$$

where $P\left(Z_{t}|X_{t}^{i}\right)∼N\left(Z_{t}-HX_{t}^{i},\Sigma_{o}\right)$. The map information can be used in the probability $P(X_{t}^{i}|X_{t-1}^{i})$ in the following:

$$P\left(X_{t}^{i}|X_{t-1}^{i}\right)=\left\{\begin{array}{cc} P_{m} & \mathrm{if}\;a\;\mathrm{particle}\;\mathrm{crossed}\;a\;\mathrm{wall}\\ 1-P_{m} & \mathrm{if}\;a\;\mathrm{particle}\;\mathrm{did}\;\mathrm{not}\;\mathrm{cross}\;a\;\mathrm{wall}, \end{array}\right.$$

where $P_{m}$ simply set to zero because human cannot pass the wall.

**Figure 13:**
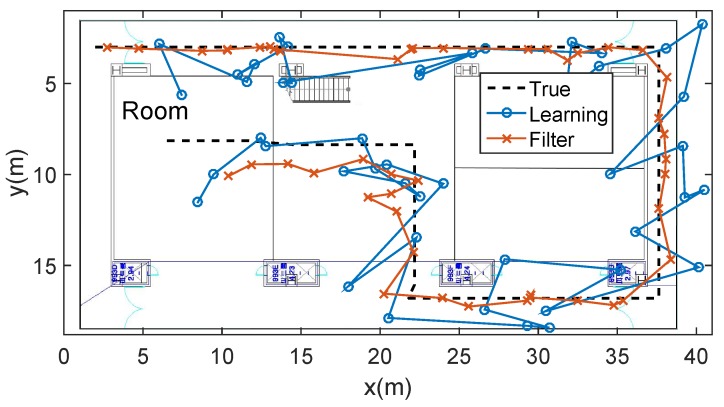
Combination of the particle filter and the learning algorithm, given map information. The test data is obtained through the dotted line. Blue circle line is the result when using only learning localization. Red cross line is the result when we combine the learning with the filter, illustrating a smoother and more accurate trajectory.

Evaluation of the particle filter based on the learning algorithm is shown in Figure 13. It is assumed that the user walks constant speed along the designated path. By the records of timestamp using the smartphone, we calculate the average 5.2 m localization error when using only learning method, and 2.9 m localization error when combining the particle filtering.

## 6. Conclusions

This paper proposes a new semi-supervised learning algorithm by combining core concepts of LapERLS’s pseudolabeling, time-series learning, and LapLS-based balancing optimization. From the indoor localization experiment, the suggested algorithm achieves high accuracy by using only a small amount of the labeled training data in comparison with the other semi-supervised algorithms. Also, this paper provides a guidance and an analysis for selecting all the parameters used in the proposed algorithm. In addition to the Wi-Fi RSSI-based localization, there are various types of indoor localization methods using IMU, Bluetooth, UWB (Ultrawide band) and camera. This paper has shown the combination with the particle filter from which expandability to involve other types of information is validated. Thus, it is expected that a fusion algorithm with the other positioning approaches might improve localization accuracy for future work.

## Figures and Tables

**Table 1:** Selected parameter values.

Value of λ	Value of $\mu_{1}$	Value of $\mu_{2}$	Value of C	Value of $C^{*}$	Value of $\sigma_{k}$
10	5	2	10	1	30
